# Zinc deficiency and risk of intracerebral hemorrhage: a retrospective cohort study

**DOI:** 10.3389/fnut.2025.1660475

**Published:** 2025-10-13

**Authors:** I-Wen Chen, Hsiu-Lan Weng, Chun-Ning Ho, Shu-Wei Liao, Yi-Chen Lai, Jheng-Yan Wu, Kuo-Chuan Hung

**Affiliations:** ^1^Department of Anesthesiology, Chi Mei Medical Center, Liouying, Tainan, Taiwan; ^2^Department of Anesthesiology, E-Da Hospital, I-Shou University, Kaohsiung, Taiwan; ^3^Department of Anesthesiology, Chi Mei Medical Center, Tainan, Taiwan; ^4^Center of General Education, Chia Nan University of Pharmacy and Science, Tainan, Taiwan; ^5^Department of Nutrition, Chi Mei Medical Center, Tainan, Taiwan

**Keywords:** zinc deficiency, intracerebral hemorrhage, pneumonia, mortality, blood–brain barrier

## Abstract

**Background:**

Intracerebral hemorrhage (ICH) accounts for 10–20% of all strokes but contributes disproportionately to stroke-related mortality and disability. Zinc, an essential trace element crucial for vascular integrity and antioxidant defense, may influence cerebrovascular health through mechanisms affecting endothelial function and blood–brain barrier stability. However, no large-scale longitudinal study has examined the association between zinc deficiency and ICH risk.

**Methods:**

We conducted a retrospective cohort study using the TriNetX Research Network database, including adults who underwent serum zinc testing between 2010 and 2023. Patients were categorized into zinc deficiency (serum zinc <70 μg/dL) and control groups (70–120 μg/dL). After applying exclusion criteria and 1:1 propensity score matching based on demographics, comorbidities, medications, and laboratory values, we analyzed the association between zinc deficiency and 12-month outcomes, including ICH, mortality, pneumonia, poor blood pressure control, and major adverse cardiac events (MACEs), using Cox proportional hazards regression.

**Results:**

The final matched cohort included 147,302 patients (73,651 per group). Zinc-deficient patients demonstrated a significantly elevated risk of ICH [hazard ratio (HR): 1.75, 95% confidence interval (CI): 1.35–2.25, *p* < 0.001], all-cause mortality (HR: 1.90, 95% CI: 1.77–2.03, *p* < 0.001), pneumonia (HR: 1.50, 95% CI: 1.40–1.60, *p* < 0.001), poor blood pressure control (HR: 1.26, 95% CI: 1.20–1.32, *p* < 0.001), and MACEs (HR: 1.12, 95% CI: 1.07–1.18, *p* < 0.001). A clear dose–response relationship was observed, with severe zinc deficiency (<50 μg/dL) conferring a greater ICH risk (HR: 2.44, 95% CI: 1.50–3.95, *p* < 0.001). The ICH association remained consistent across patient subgroups, with no significant effect modification. Multivariate analysis confirmed zinc deficiency as an independent ICH predictor (adjusted HR: 1.87, 95% CI: 1.53–2.29, *p* < 0.001).

**Conclusion:**

Zinc deficiency is a novel, independent, and potentially modifiable risk factor for ICH. The dose-dependent relationship and consistency across patient populations supports biological plausibility. These findings suggest that routine zinc assessment and targeted supplementation in deficient patients may offer new opportunities for ICH prevention, warranting prospective intervention trials to establish causality and optimal therapeutic strategies.

## 1 Introduction

Intracerebral hemorrhage (ICH) represents one of the most devastating forms of stroke, accounting for approximately 10–20% of all strokes but contributing disproportionately to stroke-related mortality and long-term disability, with up to 40% of patients dying within the first month (1–4). Beyond its immediate lethality, ICH imposes substantial healthcare burdens, with survivors often requiring extensive rehabilitation and long-term care due to severe neurological deficits, including paralysis, speech impairment, and cognitive dysfunction (5–8). Understanding ICH risk becomes particularly crucial because, unlike ischemic stroke, where effective acute interventions exist, therapeutic options for ICH remain extremely limited once bleeding occurs (2). Traditional risk factors for ICH include hypertension (the most significant contributor), advanced age, anticoagulant and antiplatelet therapy, arteriovenous malformations, and lifestyle factors such as excessive alcohol consumption and cocaine use (9–11). However, these established factors do not fully explain the variability in ICH occurrence across different populations, suggesting that additional modifiable risk factors may contribute to hemorrhagic stroke susceptibility, highlighting the critical need to identify novel preventable causes.

Zinc, an essential trace element involved in over 300 enzymatic reactions, has emerged as a compelling candidate given its crucial roles in maintaining vascular integrity, immune function, and neurological health (12–14). Evidence suggests that zinc plays a multifaceted role in vascular biology, particularly in preserving microvascular stability (15–17). Its deficiency may compromise endothelial integrity, increasing susceptibility to vessel rupture and structural degeneration, which are pathological processes fundamental to the onset of hemorrhagic stroke. While previous research has explored the role of zinc in stroke, most studies have focused predominantly on ischemic stroke, particularly in relation to dietary zinc intake (18, 19). Although a small case–control study by Karadas et al. found that serum zinc levels were significantly lower in patients with acute hemorrhagic stroke than in healthy controls (20), such findings are based on small sample sizes and a cross-sectional design, making it difficult to establish temporality or causality.

Despite growing interest in the role of zinc in vascular health, no robust longitudinal cohort study has definitively examined its independent association with intracerebral hemorrhage. This knowledge gap represents a significantly missed opportunity to identify a potentially modifiable risk factor that could inform both prevention strategies and clinical management approaches. Therefore, we conducted this retrospective cohort study using a large, diverse healthcare database to investigate whether zinc deficiency independently increases the risk of ICH and to explore potential dose–response relationships and effect modifications across different patient populations.

## 2 Methods

### 2.1 Study design and data source

This retrospective cohort study utilized the TriNetX Research Network, a global federated health research platform that provides access to de-identified electronic health records (EHRs) from healthcare organizations across multiple countries, including the United States, United Kingdom, Germany, Italy, Spain, Brazil, India, and other regions. Most of the study population in the TriNetX database was from the United States, with smaller contributions from other countries. The TriNetX platform aggregates comprehensive clinical data, such as demographics, diagnoses, procedures, medications, and laboratory values, from a diverse range of healthcare systems, enabling large-scale epidemiological research while maintaining patient privacy through robust de-identification methods. Numerous clinical studies have been published using the TriNetX database (21–23), demonstrating its reliability and validity for real-world research. The study protocol was approved by the Institutional Review Board of Chi Mei Medical Center (IRB number: 11310-E04), which granted a waiver of informed consent owing to the retrospective nature of the study involving secondary analysis of pre-existing, de-identified data without any direct patient contact or intervention.

### 2.2 Study population

We included adults (≥18 years) who underwent serum zinc testing from January 1, 2010, to December 31, 2023. The broad inclusion window was designed to enhance statistical power and capture long-term clinical outcomes. The date of zinc concentration testing served as the index date, establishing the temporal baseline for all subsequent event tracking and outcome evaluations. Patients were categorized into two groups based on their serum zinc levels: the zinc deficiency group included patients with serum zinc concentrations below 70 μg/dL, while the control group comprised patients with zinc levels between 70 and 120 μg/dL, representing the normal physiological range (24, 25).

### 2.3 Exclusion criteria

To enhance the validity of our findings and reduce confounding from acute or unrelated medical conditions, we applied a series of targeted exclusion criteria. First, we excluded all individuals with a documented history of ICH before the index date to restrict the analysis to incident cases and to avoid bias from pre-existing disease. Patients with stage 4–5 chronic kidney disease or end-stage renal disease were also excluded, given their known impact on zinc metabolism and their distinct pathophysiological profiles (26, 27), which could confound the relationship between zinc deficiency and hemorrhagic risk.

To avoid reverse causation, we further excluded patients who had experienced sepsis, ICU admission, acute kidney injury, intracranial trauma, or COVID-19 infection within 1 month prior to zinc testing, as these acute conditions may transiently alter zinc levels. Lastly, we excluded patients with a history of cerebral infarction, large-artery occlusion, transient ischemic attack, cerebral artery dissection, aneurysm, arteriovenous malformation, or brain tumor, each of which represents alternative etiologies for intracranial hemorrhage that could obscure zinc-specific associations.

### 2.4 Data collection and propensity score matching

Baseline characteristics and comorbidities were extracted from the three-year period preceding the index date. To minimize confounding and create comparable groups, we employed a 1:1 propensity score matching approach using a greedy nearest-neighbor algorithm. The matching algorithm incorporated essential demographic variables, including age, sex, race, and body mass index, along with medication use patterns, such as antihypertensive agents, antidiabetic drugs, anticoagulants, and antiplatelet therapies. We also matched zinc supplementation status and several laboratory parameters, including estimated glomerular filtration rate, serum albumin, hemoglobin A1c, and hemoglobin levels. To address potential nutritional confounding, we additionally matched patients for the presence of malnutrition and other nutritional deficiencies (e.g., vitamin B or C), as these conditions often coexist with zinc deficiency and could independently influence hemorrhagic risk through their effects on vascular integrity and coagulation function.

Information on comorbidities, such as hypertension, obesity, vitamin D deficiency, anemia, and malnutrition, was obtained based on ICD-10-CM codes available in the TriNetX database. These diagnostic codes are routinely used in electronic health records contributed by participating healthcare organizations, ensuring a standardized approach across sites. The specific ICD-10-CM codes used for each disease are provided in Supplementary Table 1.

### 2.5 Outcome definitions

The primary outcome was new-onset ICH at the 12-month follow-up, chosen because this timeframe balances adequate exposure time with minimization of competing risks and loss to follow-up. Secondary outcomes included all-cause mortality, pneumonia, poor blood pressure control (defined as systolic blood pressure >180 mmHg or the presence of an ICD-10 code for hypertensive crisis), retinal or vitreous hemorrhage, and major adverse cardiac events (MACEs), including cardiac arrest, ventricular arrhythmias, atrial fibrillation or flutter, and myocardial infarction. We selected pneumonia as a positive control outcome based on established literature demonstrating that zinc deficiency impairs immune function and increases susceptibility to infection (28). Conversely, retinal or vitreous hemorrhage served as a negative control, as this outcome should theoretically be unrelated to the systemic zinc status, helping to validate our analytical approach. Poor blood pressure control was included to evaluate whether any observed association between zinc deficiency and ICH might be mediated by poor blood pressure control, given that hypertension represents the most common cause of spontaneous ICH (29).

### 2.6 Subgroup analysis

To examine potential effect modification across different patient populations, we conducted pre-specified subgroup analyses stratified by key clinical characteristics, including age groups (18–50 versus >50 years), sex, anemia status, hypertension, diabetes, obesity, and concurrent nutritional deficiencies. These subgroups were selected based on their clinical relevance and potential biological interactions with zinc metabolism. This approach allowed us to identify subpopulations that may be more vulnerable to zinc deficiency-related complications and to assess whether the association between zinc deficiency and ICH varied across different patient profiles.

### 2.7 Sensitivity analysis

To assess the robustness of our findings and address specific methodological concerns, we performed two sensitivity analyses. Model I excluded all patients with any history of chronic kidney disease, recognizing that chronic kidney disease frequently accompanies zinc deficiency and independently increases hemorrhagic risk through multiple mechanisms, including altered mineral metabolism, uremic bleeding tendencies, and medication effects. This study aimed to isolate the direct effect of zinc deficiency on ICH risk. Model II excluded patients using anticoagulant or antiplatelet medications because these pharmacological agents substantially increase bleeding risk and could confound the association between zinc deficiency and hemorrhagic outcomes. This analysis evaluated whether our findings remained significant in the absence of pharmacological bleeding enhancement, helping to distinguish between zinc-related and medication-related hemorrhagic risks.

### 2.8 Dose–response relationship

To explore potential dose-dependent associations between zinc deficiency severity and clinical outcomes, we examined severe zinc deficiency, defined as serum zinc levels below 50 μg/dL. This threshold represents markedly deficient zinc status and allowed us to assess whether more profound zinc deficiency conferred a greater risk for ICH and other outcomes. We applied identical analytical approaches, including propensity score matching, to ensure comparability between severely zinc-deficient patients and appropriately matched controls, thereby testing whether a biological gradient existed in the relationship between zinc deficiency severity and hemorrhagic complications.

### 2.9 Statistical analysis

Continuous variables are presented as means with standard deviations, while categorical variables are expressed as frequencies and percentages. Propensity score matching quality was assessed using standardized mean differences, with values less than 0.1 indicating adequate balance between groups, supplemented by visual inspection of propensity score distributions. Time-to-event outcomes were analyzed using Kaplan–Meier survival curves with log-rank tests for between-group comparisons. Cox proportional hazards regression models were used to calculate hazard ratios with 95% confidence intervals (CIs), appropriately handling censored data and time-varying risk. For subgroup analyses, statistical significance was evaluated using a confidence interval overlap assessment. Multivariate Cox regression analysis identified independent predictors of ICH, incorporating all clinically relevant covariates balanced through matching. Statistical analyses were performed using the TriNetX Analytics Platform, which utilizes R version 3.6.3 and integrated analytics tools within the TriNetX system. Two-sided *p*-values less than 0.05 were considered statistically significant.

## 3 Results

### 3.1 Patient selection and patient characteristics

**Figure 1:**
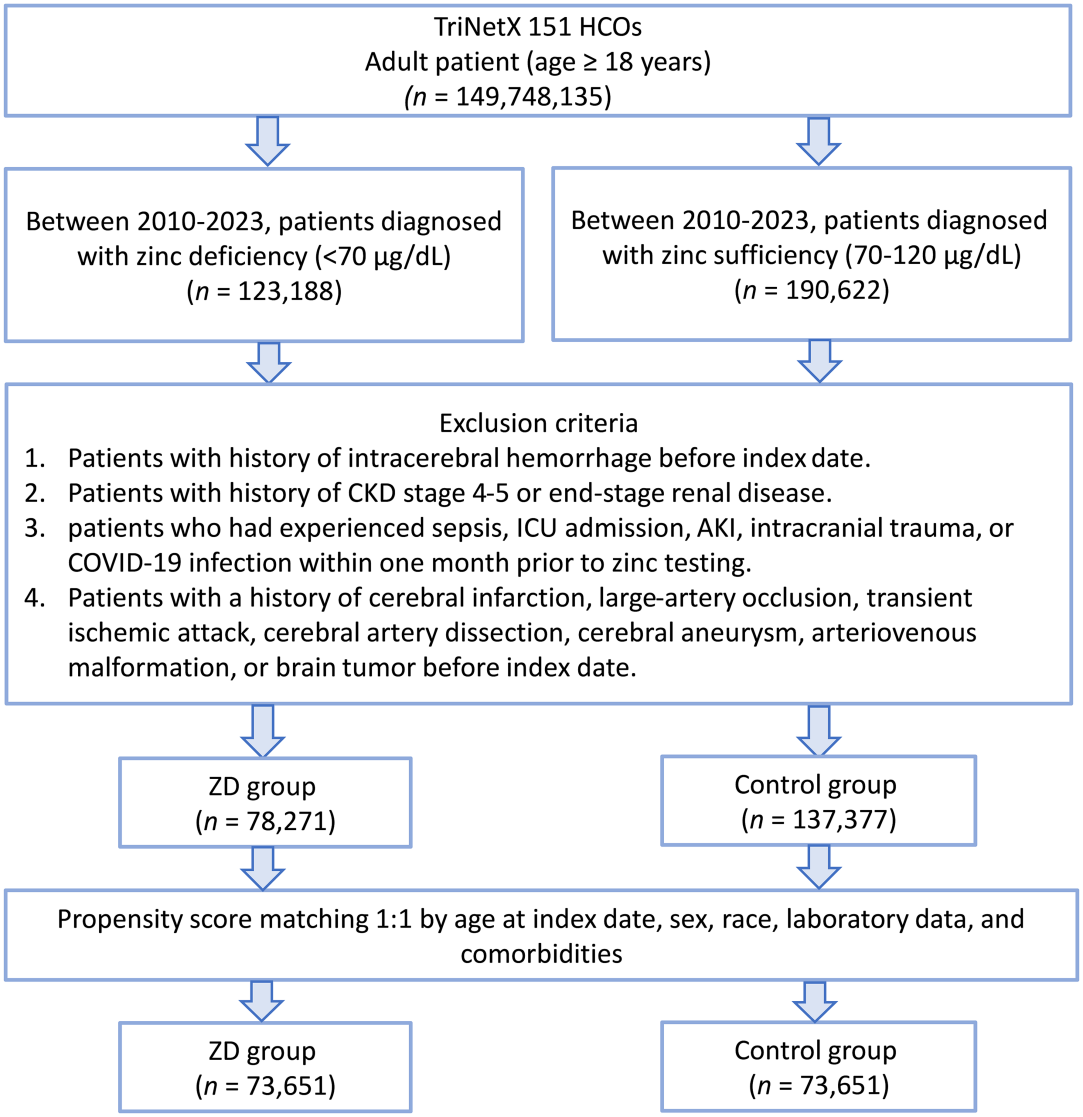
Patient selection flowchart from the TriNetX database. The flowchart illustrates the systematic exclusion process applied to identify eligible patients with zinc deficiency (ZD) and zinc sufficiency (control group). HCOs: Healthcare Organizations; CKD: chronic kidney disease; AKI: acute kidney injury; ICU: intensive care unit.

**Figure 2:**
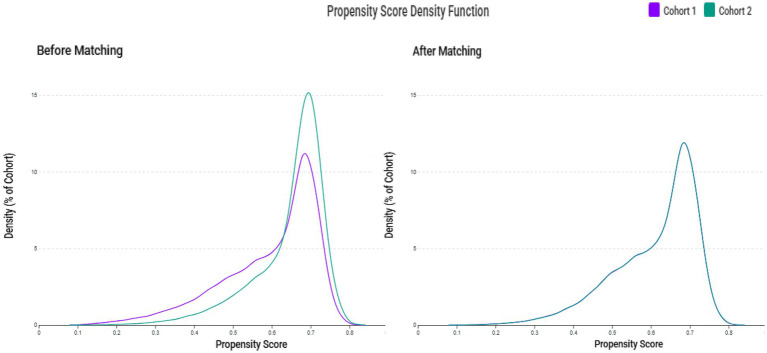
Propensity score density distributions before and after matching. The left panel shows the distribution of propensity scores between the zinc deficiency group (Cohort 1, purple) and the control group (Cohort 2, green) before matching, indicating a noticeable imbalance. The right panel shows improved overlap and covariate balance after 1:1 propensity score matching using age, sex, race, laboratory data, and comorbidities. Please note that the axis scales are automatically generated by the TriNetX Analytics Platform and cannot be manually adjusted; as such, comparisons should focus on the shape and overlap of the curves rather than the absolute scale of the axes.

A total of 149,748,135 adult patients from 151 healthcare organizations were screened. Between 2010 and 2023, 123,188 patients were identified as zinc-deficient and 190,622 as zinc-sufficient (Figure 1). After excluding patients with prior ICH, advanced CKD, recent sepsis, ICU admission, AKI, intracerebral trauma, COVID-19, or other cerebral diseases (e.g., infarction, aneurysm, or brain tumor), 78,271 zinc-deficient and 137,377 zinc-sufficient patients remained. Propensity score matching (1:1) was performed using patient demographics, laboratory values, and comorbidities, resulting in 73,651 well-balanced patients per group for the final analysis. The distribution of propensity scores showed substantial imbalance before matching but achieved excellent overlap between groups after matching (Figure 2), confirming successful covariate balance and comparability for outcome analysis.

Before matching, patients with zinc deficiency differed significantly from controls in several characteristics, including age (47.0 ± 19.0 vs. 44.8 ± 18.0), comorbidities, renal function, and medication use (e.g., anticoagulants) (Table 1). For example, patients with ZD had higher rates of hypertension, neoplasms, chronic lower respiratory diseases, anemia, liver disease, nicotine dependence, malnutrition, and ischemic heart diseases, as well as more frequent use of anticoagulants and insulin. After matching, 73,651 patients remained in each group, with well-balanced baseline characteristics (all SMDs <0.1). The matched cohorts showed no meaningful differences in demographics, comorbidities, or relevant laboratory and medication variables, thus ensuring comparability in outcome assessment.

**Table 1:** Baseline characteristics of patients before and after propensity score matching.

Variables	Before matching		After matching			
	ZD group (*n* = 78,271)	Control group (*n* = 137,377)	SMD†	ZD group (*n* = 73,651)	Control group (*n* = 73,651)	SMD†
Patient characteristics						
Age at index (years)§	47.0 ± 19.0	44.8 ± 18.0	0.121	46.5 ± 19.0	46.1 ± 18.3	0.021
Female	55,003 (70.3%)	93,066 (67.7%)	0.055	52,017 (70.6%)	53,430 (72.5%)	0.043
BMI kg/m^2^	29.7 ± 10.0	30.0 ± 9.5	0.029	29.9 ± 9.9	30.1 ± 9.8	0.022
White	53,422 (68.3%)	98,001 (71.3%)	0.067	50,716 (68.9%)	50,403 (68.4%)	0.009
Comorbidities						
Other nutritional deficiencies¶	17,939 (22.9%)	26,134 (19.0%)	0.096	16,110 (21.9%)	16,286 (22.1%)	0.006
Essential (primary) hypertension	18,155 (23.2%)	24,901 (18.1%)	0.125	15,976 (21.7%)	16,022 (21.8%)	0.002
Overweight and obesity	17,131 (21.9%)	25,976 (18.9%)	0.074	15,939 (21.6%)	16,240 (22.1%)	0.010
Neoplasms	15,457 (19.7%)	21,740 (15.8%)	0.103	13,715 (18.6%)	13,921 (18.9%)	0.007
Disorders of lipoprotein metabolism and other lipidemias	13,609 (17.4%)	22,965 (16.7%)	0.018	12,588 (17.1%)	12,557 (17.0%)	0.001
Vitamin D deficiency	13,070 (16.7%)	20,020 (14.6%)	0.059	11,921 (16.2%)	12,107 (16.4%)	0.007
Chronic lower respiratory diseases	10,912 (13.9%)	14,444 (10.5%)	0.105	9,444 (12.8%)	9,551 (13.0%)	0.004
Anemias	11,071 (14.1%)	10,763 (7.8%)	0.203	8,727 (11.8%)	8,632 (11.7%)	0.004
Diabetes mellitus	9,384 (12.0%)	12,454 (9.1%)	0.095	8,227 (11.2%)	8,229 (11.2%)	<0.001
Diseases of liver	8,388 (10.7%)	8,057 (5.9%)	0.177	6,346 (8.6%)	6,264 (8.5%)	0.004
Nicotine dependence	5,980 (7.6%)	6,061 (4.4%)	0.136	4,615 (6.3%)	4,596 (6.2%)	0.001
Malnutrition	6,138 (7.8%)	4,542 (3.3%)	0.199	4,121 (5.6%)	4,072 (5.5%)	0.003
Ischemic heart diseases	4,582 (5.9%)	5,015 (3.7%)	0.104	3,719 (5.0%)	3,673 (5.0%)	0.003
Other cardiac arrhythmias	3,860 (4.9%)	4,784 (3.5%)	0.072	3,230 (4.4%)	3,294 (4.5%)	0.004
COVID-19	2,850 (3.6%)	4,526 (3.3%)	0.019	2,630 (3.6%)	2,635 (3.6%)	<0.001
Chronic kidney disease (CKD)	2,832 (3.6%)	2,743 (2.0%)	0.098	2,231 (3.0%)	2,176 (3.0%)	0.004
Heart failure	3,035 (3.9%)	2,464 (1.8%)	0.126	2,220 (3.0%)	2,159 (2.9%)	0.005
Alcohol related disorders	3,549 (4.5%)	2,366 (1.7%)	0.162	2,181 (3.0%)	2,113 (2.9%)	0.005
Atrial fibrillation and flutter	2,646 (3.4%)	2,456 (1.8%)	0.101	1997 (2.7%)	2015 (2.7%)	0.002
Long term use of anticoagulants and antithrombotics/antiplatelets	2,269 (2.9%)	2,171 (1.6%)	0.089	1819 (2.5%)	1800 (2.4%)	0.002
Atherosclerosis	1,213 (1.6%)	1,141 (0.8%)	0.066	906 (1.2%)	891 (1.2%)	0.002
Cerebrovascular diseases	745 (1.0%)	780 (0.6%)	0.044	583 (0.8%)	590 (0.8%)	0.001
Laboratory data						
Hemoglobin≥12 mg/dL†	49,565 (63.3%)	88,503 (64.4%)	0.023	46,781 (63.5%)	47,253 (64.2%)	0.013
Hemoglobin A1c ≥ 7%‡	4,496 (5.7%)	6,530 (4.8%)	0.044	4,056 (5.5%)	4,002 (5.4%)	0.003
Albumin g/dL (≥3.5 g/dL)	45,582 (58.2%)	75,128 (54.7%)	0.072	42,412 (57.6%)	43,668 (59.3%)	0.035
eGFR>60 mL/min/1.73 m^2^	52,097 (66.6%)	84,502 (61.5%)	0.105	47,927 (65.1%)	47,924 (65.1%)	<0.001
Medications						
Cardiovascular medications	39,932 (51.0%)	55,537 (40.4%)	0.214	35,724 (48.5%)	35,938 (48.8%)	0.006
Anticoagulants	21,191 (27.1%)	22,663 (16.5%)	0.258	17,716 (24.1%)	17,753 (24.1%)	0.001
Antilipemic agents	10,892 (13.9%)	16,522 (12.0%)	0.056	9,882 (13.4%)	9,856 (13.4%)	0.001
Platelet aggregation inhibitors	7,975 (10.2%)	9,621 (7.0%)	0.114	6,789 (9.2%)	6,785 (9.2%)	<0.001
Insulins and analogs	8,107 (10.4%)	8,729 (6.4%)	0.145	6,721 (9.1%)	6,691 (9.1%)	0.001
Blood glucose lowering drugs	7,216 (9.2%)	10,736 (7.8%)	0.050	6,574 (8.9%)	6,539 (8.9%)	0.002
Zinc supplementation	2,248 (2.9%)	3,299 (2.4%)	0.029	2022 (2.7%)	2019 (2.7%)	<0.001

BMI, body mass index; SMD, standardized mean differences; †SMD values <0.1 indicate adequate balance between groups; eGFR: estimated Glomerular Filtration Rate; ¶ Other nutritional deficiencies included vitamin A deficiency, thiamine deficiency, niacin deficiency, deficiency of other B group vitamins, ascorbic acid deficiency, dietary calcium deficiency, or dietary selenium deficiency; §After matching, ZD group vs. control group (age distribution): 18–50 years: 54.6% vs. 55.3%; >50 years: 45.4% vs. 44.7%. †After matching, ZD group vs. control group (hemoglobin): 12.5 ± 2.1 mg/dL vs. 12.8 ± 1.9 mg/dL. ‡After matching, ZD group vs. control group (HbA1c): 5.7 ± 1.2% vs. 5.7 ± 1.1%.

### 3.2 Association between zinc deficiency and 1-year outcomes

**Figure 3:**
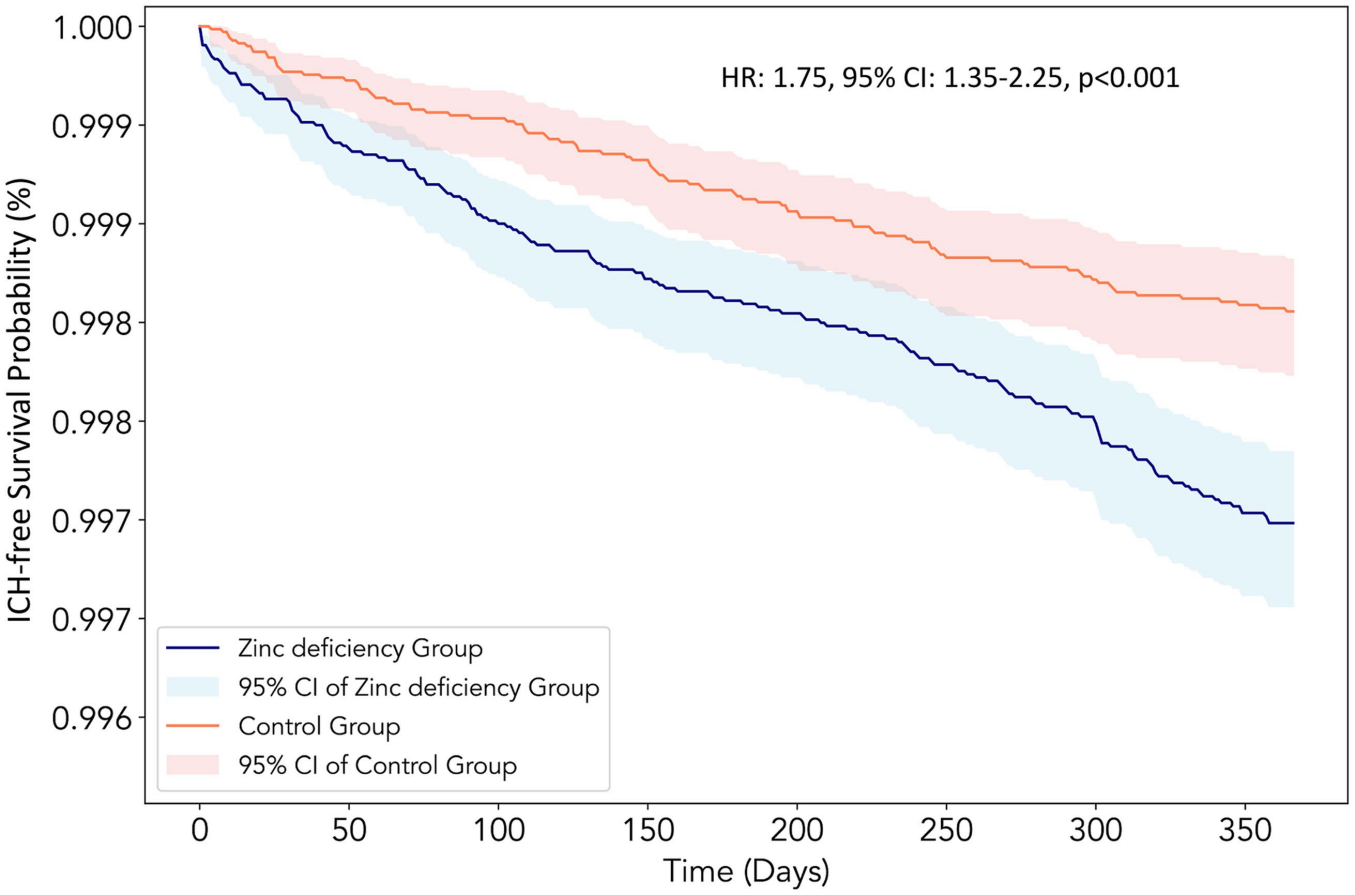
Kaplan–Meier survival curves for intracerebral hemorrhage (ICH)-free survival at the 12-month follow-up. The curves compare patients with Zinc deficiency (blue line, *n* = 73,651) with control patients with normal Zinc levels (orange line, *n* = 73,651) after propensity score matching. The y-axis represents the probability of remaining free from intracerebral hemorrhage, and the x-axis shows the time in days from the index date. The shaded areas represent the 95% confidence intervals for each group. The Zinc deficiency group demonstrated a significantly lower ICH-free survival probability throughout the follow-up period [hazard ratio (HR):1.75, 95% confidence interval (CI): 1.35–2.25, *p* < 0.001], consistent with a 75% increased hazard ratio for developing intracerebral hemorrhage.

Zinc-deficient patients demonstrated a substantially elevated risk of ICH at the 12-month follow-up (HR:1.75, 95% CI: 1.35–2.25, *p* < 0.001) compared to zinc-sufficient controls (Table 2), suggesting a 75% increased risk of developing this serious cerebrovascular complication. Kaplan–Meier survival analysis revealed a clear separation between the two groups throughout the 12-month follow-up period (Figure 3). The zinc-deficient group exhibited a consistently lower ICH-free survival probability than the controls, with the survival curves beginning to diverge early in the follow-up period and maintaining separation throughout the entire observation window.

**Table 2:** Association between zinc deficiency and 1-year outcomes.

Outcomes	ZD group (*n* = 73,651)	Control group (*n* = 73,651)	HR (95% CI)	*p*-value
	Events (%)	Events (%)		
ICH	161 (0.22%)	95 (0.13%)	1.75 (1.35–2.25)	<0.001
Mortality	2,346 (3.2%)	1,272 (1.7%)	1.90 (1.77–2.03)	<0.001
MACEs	3,968 (5.4%)	3,624 (4.9%)	1.12 (1.07–1.18)	<0.001
Poor blood pressure control	3,484 (4.7%)	2,857 (3.9%)	1.26 (1.20–1.32)	<0.001
Pneumonia	2,150 (2.9%)	1,478 (2.0%)	1.50 (1.40–1.60)	<0.001
Retinal/vitreous hemorrhage	81 (0.11%)	101 (0.14%)	0.82 (0.62–1.10)	0.193

ICH, intracerebral hemorrhage; MACEs, major adverse cardiac events; ZD, zinc deficiency; HR, hazard ratio; CI, confidence interval.

The association between zinc deficiency and mortality was even more pronounced (HR:1.90, 95% CI: 1.77–2.03, *p* < 0.001), indicating an almost doubled mortality risk within the first year. Beyond these outcomes, zinc deficiency has emerged as a significant predictor of cardiopulmonary complications. Patients exhibited a 50% higher likelihood of developing pneumonia (HR: 1.50, 95% CI: 1.40–1.60, *p* < 0.001), supporting the established connection between zinc status and immune function. The risk of poor blood pressure control increased by 26% (HR: 1.26, 95% CI: 1.20–1.32, *p* < 0.001), while major adverse cardiac events showed a more modest but statistically significant 12% elevation (HR: 1.12, 95% CI: 1.07–1.18, *p* < 0.001). Notably, retinal or vitreous hemorrhage showed no significant association with zinc status (HR: 0.82, 95% CI: 0.62–1.10, *p* = 0.193), serving as an appropriate negative control outcome.

### 3.3 Association between zinc deficiency and 2-year outcomes and clinical significance

An extended follow-up of 24 months revealed persistent but somewhat attenuated associations across most outcomes (Table 3). Patients with zinc deficiency faced a 44% greater risk of developing ICH (HR: 1.44; 95% CI: 1.18–1.75; *p* < 0.001), highlighting the potential long-term neurological consequences associated with micronutrient imbalance. Mortality risk demonstrated remarkable consistency over the extended timeframe (HR:1.80, 95% CI: 1.70–1.91, *p* < 0.001) at 2 years. The persistence of this 80% increase in mortality risk underscores the profound clinical implications of zinc deficiency as a modifiable risk factor. Pneumonia risk remained substantially elevated (HR: 1.42, 95% CI: 1.34–1.50, *p* < 0.001), while the risks of poor blood pressure control and major cardiac events showed similar patterns to the one-year analysis.

**Table 3:** Association between zinc deficiency and 2-year outcomes.

Outcomes	ZD group (*n* = 73,651)	Control group (*n* = 73,651)	HR (95% CI)	*p*-value
	Events (%)	Events (%)		
ICH	236 (0.32%)	171 (0.23%)	1.44 (1.18–1.75)	< 0.001
Mortality	3,313 (4.5%)	1911 (2.6%)	1.80 (1.70–1.91)	< 0.001
MACEs	5,207 (7.1%)	4,781 (6.5%)	1.13 (1.08–1.17)	< 0.001
Poor blood pressure control	4,724 (6.4%)	4,077 (5.5%)	1.21 (1.16–1.26)	< 0.001
Pneumonia	3,100 (4.2%)	2,284 (3.1%)	1.42 (1.34–1.50)	< 0.001
Retinal/vitreous hemorrhage	129 (0.18%)	140 (0.19%)	0.96 (0.75–1.22)	0.714

ICH, intracerebral hemorrhage; MACEs, major adverse cardiac events; ZD, zinc deficiency; HR, hazard ratio; CI, confidence interval.

### 3.4 Sensitivity analysis

Sensitivity analyses provided crucial insights into the robustness of the association between zinc deficiency association. When patients with anticoagulant or antiplatelet therapy were excluded (Model I) (Table 4), the risk for ICH increased dramatically to 4.4 (95% CI: 2.46–7.86, *p* < 0.001). This fourfold risk elevation suggests that zinc deficiency may exert independent hemorrhagic effects beyond pharmacological bleeding enhancement, potentially through mechanisms involving vascular integrity or coagulation function.

**Table 4:** Sensitivity analysis of association between zinc deficiency and 1-year outcomes.

Outcomes	Model I		Model II	
	HR (95% CI)	*p*-values	HR (95% CI)	*p*-values
ICH	4.4 (2.46–7.86)	< 0.001	1.81 (1.44–2.28)	< 0.001
Mortality	2.24 (1.99–2.53)	< 0.001	2.11 (1.97–2.26)	< 0.001
MACEs	1.08 (0.99–1.16)	0.051	1.19 (1.14–1.24)	< 0.001
Poor blood pressure control	1.25 (1.15–1.36)	< 0.001	1.29 (1.22–1.35)	< 0.001
Pneumonia	1.34 (1.18–1.52)	< 0.001	1.59 (1.49–1.69)	< 0.001
Retinal/vitreous hemorrhage	1.03 (0.67–1.57)	0.910	1.00 (0.74–1.36)	0.994

Model I: patients with previous history of anticoagulation/antiplatelet use were excluded in both group (*n* = 56,407 for each group) Model II: patients with previous history of CKD were excluded in both group (*n* = 82,834 for each group). ICH, intracerebral hemorrhage; MACEs, major adverse cardiac events; ZD, zinc deficiency; HR, hazard ratio; CI, confidence interval.

Excluding patients with chronic kidney disease (Model II) resulted in a comparable elevated risk of ICH (HR: 1.81; 95% CI: 1.44–2.28; *p* < 0.001) (Table 4). This finding indicates that the association persisted even after removing patients with conditions known to affect both zinc metabolism and bleeding risk. These sensitivity analyses strengthen confidence in the primary findings while highlighting that the hemorrhagic risk of zinc deficiency may be most pronounced in patients not receiving anticoagulation therapy, where the relative contribution of zinc-related mechanisms becomes more apparent.

### 3.5 Dose–response relationship

Analysis of severe zinc deficiency (serum zinc <50 μg/dL) revealed a clear dose–response relationship, supporting biological plausibility (Table 5). Patients with severe deficiency demonstrated a hazard ratio of 2.44 (95% CI: 1.50–3.95, *p* < 0.001) for ICH, representing more than double the risk observed with moderate deficiency. This dose-dependent association strengthens the argument for a causal relationship between zinc status and hemorrhagic complications.

**Table 5:** Impact of severe zinc deficiency on 1-year outcomes.

Outcomes	ZD group (*n* = 8,507)	Control group (*n* = 8,507)	HR (95% CI)	*p*-value
	Events (%)	Events (%)		
ICH	52 (0.61%)	24 (0.28%)	2.44 (1.50–3.95)	< 0.001
Mortality	1,132 (13.3%)	482 (5.7%)	2.60 (2.34–2.90)	< 0.001
MACEs	889 (10.5%)	786 (9.2%)	1.23 (1.12–1.36)	< 0.001
Poor blood pressure control	685 (8.1%)	627 (7.4%)	1.20 (1.08–1.34)	0.001
Pneumonia	648 (7.6%)	439 (5.2%)	1.64 (1.45–1.85)	< 0.001
Retinal/vitreous hemorrhage	15 (0.18%)	23 (0.27%)	0.72 (0.38–1.39)	0.329

ICH, intracerebral hemorrhage; MACEs, major adverse cardiac events; ZD, zinc deficiency; HR, hazard ratio; CI, confidence interval.

The mortality risk among severely zinc-deficient patients proved particularly striking, with a hazard ratio of 2.60 (95% CI: 2.34–2.90, *p* < 0.001), indicating a 160% increased risk of death. Major adverse cardiac events also showed dose-dependent increases (HR: 1.23, 95% CI: 1.12–1.36, *p* < 0.001), while pneumonia risk reached 1.64 (HR: 1.64, 95% CI: 1.45–1.85, *p* < 0.001). These findings suggest that the severity of zinc deficiency correlates directly with the magnitude of clinical risk, providing important guidance for prioritizing intervention strategies.

### 3.6 Subgroup analysis for 1-year intracerebral hemorrhage risk

Subgroup analyses revealed remarkable consistency in the association between zinc deficiency and ICH across diverse patient populations (Table 6). Gender-stratified analysis showed similar effect sizes for both males (HR: 1.71, 95% CI: 1.19–2.46, *p* = 0.003) and females (HR: 1.67, 95% CI: 1.15–2.41, *p* = 0.007), with no significant interaction (*p* = 0.930). Age stratification demonstrated comparable risks between younger adults aged 18–50 years (HR: 1.90, 95% CI: 1.01–3.55, *p* = 0.042) and older patients (HR: 1.67, 95% CI: 1.26–2.22, *p* < 0.001), with no significant effect modification by age (*p* = 0.740).

**Table 6:** Subgroup analyses of association between zinc deficiency and risk of intracranial hemorrhage at 12-month Follow-Up.

Subgroup analysis	Number of each group	HR (95% CI)	*p*-value	*p* for interaction
Sex				
Male	20,203	1.71 (1.19–2.46)	0.003	Reference
female	52,139	1.67 (1.15–2.41)	0.007	0.930
Age				
18–50 years	36,165	1.90 (1.01–3.55)	0.042	Reference
>50 years	36,122	1.67 (1.26–2.22)	<0.001	0.740
Anemia				
Yes	12,372	1.60 (1.02–2.54)	0.041	Reference
No	60,173	1.79 (1.32–2.45)	<0.001	0.694
Hypertension				
Yes	18,435	1.60 (1.10–2.32)	0.013	Reference
No	54,189	1.74 (1.22–2.48)	0.002	0.754
DM				
Yes	9,698	1.74 (1.00–3.03)	0.046	Reference
No	62,978	1.67 (1.25–2.24)	<0.001	0.903
Obesity				
Yes	19,152	1.57 (0.87–2.85)	0.130	Reference
No	53,283	1.68 (1.25–2.25)	<0.001	0.846
Other nutrition deficiency				
Yes	24,120	1.64 (1.10–2.46)	0.016	Reference
No	48,455	1.83 (1.30–2.56)	<0.001	0.688

HR, hazard ratio; CI, confidence interval; DM, diabetes mellitus; *p* < 0.05 was considered significance.

Stratified analyses further confirmed the association regardless of anemia, hypertension, diabetes, obesity, or other nutritional deficiencies, with all interaction *p*-values exceeding 0.05, suggesting that the relationship between zinc deficiency and hemorrhagic risk was largely independent of these clinical factors.

### 3.7 Independent risk factors for new-onset intracerebral hemorrhage

Multivariable Cox regression analysis confirmed zinc deficiency as an independent predictor of ICH, with an adjusted hazard ratio of 1.87 (95% CI: 1.53–2.29, *p* < 0.001) after controlling for multiple confounders (Table 7). This finding revealed that zinc deficiency was one of the most impactful modifiable risk factors identified in the analysis. The presence of liver disease (HR: 2.01; *p* < 0.001) and male sex (HR: 1.77; *p* < 0.001) were also identified as significant independent predictors of ICH. Additional independent risk factors included anemia (HR: 1.64, *p* < 0.001), ischemic heart disease (HR: 1.55, *p* = 0.003), malnutrition (HR: 1.48, *p* = 0.013), and nicotine dependence (HR: 1.38, *p* = 0.041). Interestingly, obesity appeared to be protective (HR: 0.70, *p* = 0.010), while traditional vascular risk factors, such as hypertension and diabetes, showed no independent association after adjustment. These findings underscore the multifactorial nature of ICH risk, with zinc deficiency emerging as a strong and modifiable contributor.

**Table 7:** Risk factors for new-onset intracranial hemorrhage.

Variable	HR (95% CI)	*P*-value
Zinc deficiency vs. control groups	1.87 (1.53, 2.29)	<0.001
Male	1.77 (1.45, 2.17)	<0.001
Age at Index	1.05 (1.04, 1.05)	<0.001
Essential (primary) hypertension	0.99 (0.78, 1.26)	0.952
Overweight and obesity	0.70 (0.53, 0.92)	0.010
Diabetes mellitus	1.03 (0.79, 1.35)	0.834
Heart failure	1.38 (0.98, 1.95)	0.065
Ischemic heart diseases	1.55 (1.16, 2.06)	0.003
Chronic kidney disease (CKD)	0.74 (0.50, 1.10)	0.142
Nicotine dependence	1.38 (1.01, 1.89)	0.041
Malnutrition	1.48 (1.09, 2.03)	0.013
Diseases of liver	2.01 (1.57, 2.56)	<0.001
Other anemias	1.64 (1.28, 2.09)	<0.001
Other nutritional deficiencies	0.88 (0.69, 1.11)	0.277
Atherosclerosis	0.79 (0.48, 1.31)	0.359

HR, hazard ratio; CI, confidence interval; †multivariate logistic regression analysis.

## 4 Discussion

This large-scale retrospective cohort study is the first to demonstrate a significant association between zinc deficiency and an increased risk of ICH in a diverse healthcare population. Our analysis of over 147,000 matched patients revealed that zinc deficiency substantially elevated the risk of ICH, with this association persisting across multiple analytical approaches and timeframes. The relationship demonstrated remarkable consistency, remaining statistically significant at both one-year and two-year follow-up periods, while showing clear dose-dependent characteristics where more severe zinc deficiency conferred progressively higher hemorrhagic risk. Most importantly, our findings proved robust across diverse patient subgroups and remained significant even after adjustment for multiple confounding variables, suggesting that zinc deficiency represents an independent and potentially modifiable risk factor for this devastating neurological complication.

Zinc serves as a critical cofactor for numerous enzymes involved in antioxidant defense, including superoxide dismutase and catalase, whose dysfunction during zinc deficiency leads to increased oxidative stress and subsequent endothelial damage (30, 31). This micronutrient also plays an essential role in maintaining tight junction integrity between endothelial cells, with deficiency states compromising the blood–brain barrier (32, 33) and potentially predisposing cerebral vessels to rupture under elevated pressures. Animal studies have provided mechanistic support for these pathways, demonstrating that zinc deficiency exacerbates vascular inflammation and oxidative stress, while promoting apoptosis of vascular smooth muscle cells and activating pro-inflammatory NF-κB signaling in endothelial cells (34–36). These experimental findings collectively illustrate how zinc deficiency creates a pro-inflammatory, oxidatively stressed vascular environment that predisposes multiple vessel types to structural weakness and functional impairment. Previous studies have reported inverse associations between zinc intake or serum levels and ischemic stroke incidence (18, 37). However, these studies were largely cross-sectional or case-cohort in design and focused exclusively on ischemic mechanisms, limiting their relevance to hemorrhagic events. Our longitudinal approach specifically addressed this knowledge gap by examining hemorrhagic rather than ischemic outcomes, providing crucial evidence that zinc deficiency may influence cerebrovascular health through mechanisms distinct from those affecting thrombotic events.

In the current study, the elevated hemorrhagic risk observed at 1 year persisted into the second year of follow-up, though with some attenuation, suggesting that zinc deficiency may exert lasting effects on vascular integrity. Perhaps the most striking is the magnitude of the association between zinc deficiency and ICH compared to other cardiovascular complications within the same cohort. Although zinc deficiency was linked to a 26% increased risk of poor blood pressure control, its association with ICH was markedly stronger, with a 75% increased risk. This disparity suggests that the influence of zinc deficiency on hemorrhagic stroke extends beyond blood pressure dysregulation, implicating deeper disruptions in cerebrovascular integrity and hemostatic balance. The inclusion of retinal or vitreous hemorrhage as a negative control outcome further strengthens our findings, as this ocular bleeding showed no association with zinc status, indicating that our observed ICH association is not simply due to a generalized bleeding tendency, but rather reflects specific cerebrovascular vulnerability. This mechanistic specificity indicates that zinc deficiency may affect cerebral vessels through distinct pathways not shared with other vascular territories, potentially involving the unique structure of the blood–brain barrier and heightened vulnerability of cerebral arterioles to pressure-induced rupture.

The clear dose–response relationship observed between zinc deficiency severity and ICH risk provides perhaps the strongest evidence for biological causality in our study. Patients with severe zinc deficiency demonstrated more than double the risk of hemorrhage than those with moderate deficiency, establishing a biological gradient that supports causal inference. This dose-dependent pattern extended beyond ICH to include cardiopulmonary complications, particularly pneumonia, which served as an important positive control. The elevated pneumonia risk observed in zinc-deficient patients is consistent with well-established immunological evidence highlighting the essential role of zinc in immune defense (28), thereby validating that our cohort definition and analytic approach effectively identified clinically relevant zinc deficiency. The parallel dose–response patterns for both ICH and pneumonia suggest that zinc deficiency exerts systemic effects that manifest across multiple organ systems, with the severity of deficiency directly correlating with the magnitude of clinical risk across diverse pathophysiological pathways.

The remarkable consistency of the association between zinc deficiency and ICH across all examined subgroups provides evidence that this relationship represents a fundamental biological phenomenon rather than an artifact limited to specific patient populations. The absence of significant effect modification by age, sex, anemia status, hypertension, diabetes, obesity, or other nutritional deficiencies suggests that the hemorrhagic effects of zinc deficiency operate through pathways that are largely independent of these traditional cerebrovascular risk factors. This universality has important clinical implications, indicating that zinc deficiency screening and correction might benefit diverse patient populations rather than being limited to specific high-risk groups. The consistent effect sizes across subgroups also strengthened confidence in our propensity score matching approach, demonstrating that residual confounding is unlikely to explain our observed associations. Perhaps most clinically relevant, the similar effect sizes in patients with and without traditional vascular risk factors suggest that zinc deficiency might represent an under-recognized pathway to ICH that operates independently of conventional stroke prevention strategies.

In the current study, while we proposed that zinc deficiency may increase ICH risk through effects on vascular endothelial function and the blood–brain barrier, our study was observational and did not directly assess underlying mechanisms or related biomarkers. Furthermore, our study relied on electronic health record data, which records the presence or absence of ICH based on ICD-10 codes, but does not provide information on the specific anatomical location or volume of bleeding. As a result, we were unable to perform analyses stratified by hemorrhage location or severity. This highlights the key limitations of using large-scale administrative databases in clinical research. Further studies with more detailed clinical and imaging data are needed to clarify the mechanistic pathways and impact of zinc deficiency on specific hemorrhage subtypes.

The persistence of zinc deficiency as a significant predictor of ICH in fully adjusted multivariate models established its role as an independent risk factor. This independent association is particularly noteworthy given that traditional vascular risk factors, such as hypertension and diabetes, showed diminished significance after full adjustment, suggesting that the hemorrhagic effects of zinc deficiency operate through mechanisms distinct from conventional cerebrovascular pathophysiology. The identification of liver disease, anemia, ischemic heart disease, malnutrition, and nicotine dependence as additional independent predictors underscores the multifactorial nature of ICH risk, while simultaneously positioning zinc deficiency as a novel and potentially modifiable target that could complement existing prevention strategies. Interestingly, our multivariate analysis indicated that obesity was associated with a lower risk of new-onset ICH, contrary to its established role as a cardiovascular risk factor. This phenomenon may reflect the so-called “obesity paradox,” which has been observed in various acute and chronic diseases, where higher body mass index sometimes confers unexpected protective effects (38). Further research is needed to clarify the underlying mechanisms of this observation in hemorrhagic stroke.

Several important limitations must be acknowledged when interpreting our findings and considering their clinical application. First, the retrospective observational design cannot definitively establish causality despite the strong associations observed. Second, our reliance on single zinc measurements may not accurately reflect long-term zinc status, as serum zinc levels can be influenced by acute illness, medications, inflammation, and diurnal variation, potentially leading to the misclassification of some patients’ true zinc status. Although C-reactive protein data are available in TriNetX, inconsistent availability and non-synchronized timing with zinc measurements could introduce bias; therefore, C-reactive protein was not included in our analysis. Consequently, residual confounding due to inflammation cannot be fully excluded. Third, because only individuals who underwent serum zinc testing were included, often because of clinical suspicion or comorbid conditions, our findings may not be generalizable to the broader population and could limit external validity. Additionally, we also lacked information on dietary zinc intake, as well as the dosage and duration of zinc supplementation, precluding analyses of nutritional factors and the impact of supplementation. These limitations may affect the accuracy of zinc status classification and restrict clinical recommendations for correcting zinc deficiency. Finally, although dose–response analysis using tertiles, quartiles, or spline curves would be ideal, it was not feasible because of the small number of patients with severe zinc deficiency and limited access to patient-level raw data in TriNetX.

## 5 Conclusion

This study established zinc deficiency as a potentially important and modifiable risk factor for ICH, opening new avenues for both prevention and therapeutic intervention. The consistent associations observed across diverse patient populations suggest that routine zinc assessment might be warranted in high-risk patients, particularly those with multiple traditional cerebrovascular risk factors or conditions that predispose them to zinc deficiency. Future prospective studies should examine whether zinc supplementation in deficient patients can reduce ICH incidence and explore the optimal timing, dosing, and duration of such interventions to maximize cerebrovascular protection while minimizing potential adverse effects.

## Data Availability

The raw data supporting the conclusions of this article will be made available by the authors, without undue reservation.
