# Supplementary material for: Zinc deficiency and risk of intracerebral hemorrhage: a retrospective cohort study
Source: Front Nutr. 2025 Oct 13;12:1660475. doi: 10.3389/fnut.2025.1660475 (PMC12554597; doi:10.3389/fnut.2025.1660475)
Supplement: Supplementary file 1 [file Table_1.DOCX]

**Supplemental table 1.** ICD-10-CM codes used for each variable

| F17 | Nicotine dependence |
| --- | --- |
| F10 | Alcohol related disorders |
| E40-E46 | Malnutrition |
| E66 | Overweight and obesity |
| I10 | Essential (primary) hypertension |
| E78 | Disorders of lipoprotein metabolism and other lipidemias |
| K70-K77 | Diseases of liver |
| D64 | Other anemias |
| C00-D49 | Neoplasms |
| E55 | Vitamin D deficiency |
| I50 | Heart failure |
| I20-I25 | Ischemic heart diseases |
| I60-I69 | Cerebrovascular diseases |
| U07.1 | COVID-19 |
| E08-E13 | Diabetes mellitus |
| I48 | Atrial fibrillation and flutter |
| I49 | Other cardiac arrhythmias |
| J40-J4A | Chronic lower respiratory diseases |
| N18 | Chronic kidney disease (CKD) |
| Z79.0 | Long term (current) use of anticoagulants and antithrombotics/antiplatelets |
| E50-E64 | Other nutritional deficiencies |
